# 한국 간호대학생의 디지털 리터러시와 문제해결과정의 관계에서 간호정보활용역량의 매개효과: 횡단적 조사연구

**DOI:** 10.7586/jkbns.26.021

**Published:** 2026-08-26

**Authors:** Chang Seung Park, Yeong Ju Ko

**Affiliations:** Department of Nursing, Cheju Halla University, Jeju, Korea; 제주한라대학교 간호학과

**Keywords:** Students, nursing, Computer literacy, Nursing informatics, Problem solving, 간호학생, 컴퓨터 활용 능력, 간호정보학, 문제해결

## Abstract

**Purpose:**

This study examined the effect of digital literacy on the problem-solving process among nursing students and tested whether nursing informatics competency mediated this relationship.

**Methods:**

This descriptive cross-sectional study included 119 nursing students. Data were analyzed using SPSS version 25.0 and PROCESS macro version 4.2, Model 4. The mediating effect was assessed using 5,000 bootstrap samples, and statistical significance was determined using the 95% confidence interval (CI).

**Results:**

Digital literacy was positively correlated with the problem-solving process (r = .63, *p* < .001). Digital literacy had a significant direct effect on the problem-solving process (c′= 0.33, *p* = .013), and nursing informatics competency had a significant indirect effect on this relationship (indirect effect = 0.38, 95% CI: 0.10–0.62). The direct effect of digital literacy on the problem-solving process remained significant after nursing informatics competency was included in the model, and the total effect was also significant (c = 0.71, *p* < .001).

**Conclusion:**

Digital literacy was directly and indirectly associated with the problem-solving process among nursing students, with nursing informatics competency serving as a significant mediating pathway. These findings suggest that educational strategies aimed at strengthening problem-solving abilities should address not only digital literacy but also nursing informatics competency. Future studies should test expanded models that include additional factors affecting the problem-solving process.

## 서론

### 1. 연구의 필요성

현대 사회의 급격한 정보통신기술의 발전과 4차 산업혁명의 흐름은 보건의료환경을 빠르게 변화시키고 있다. 빅데이터, 인공지능, 사물인터넷과 같은 첨단 디지털 기술이 임상현장에 빠르게 도입됨에 따라[1] 간호사는 과거에 비해 복합적이고 다양한 건강문제를 다루는 동시에 디지털 중심의 간호환경에 직면하고 있다[2]. 이러한 환경에서 간호사가 전문직 역할을 효과적으로 수행하기 위해서는 환자의 건강문제를 해결하기 위해 방대한 디지털 정보를 간호지식과 결합하여 최적의 대안을 도출해내는 문제해결과정을 갖추는 것이 필수적이다[3,4].

문제해결과정이란 간호상황에서 발생하는 문제를 인식하고, 이를 해결하기 위해 지식과 기술을 활용하여 바람직한 목표 상태에 도달하기 위한 논리적 사고 과정을 의미한다[5]. 이는 문제 인식부터 정보수집, 분석, 대안 설정, 실행 및 결과 평가에 이르는 순환적이고 단계적인 사고체계로[5], 이러한 과정 속에서 올바른 의사결정을 내리기 위해서는 다양한 경로를 통해 데이터를 탐색하고 적절히 활용할 수 있는 정보적 자질이 필요하다[6]. 특히 질병 구조의 복잡화와 환자 안전에 대한 사회적 요구가 급증하고 있는 보건의료 현장에서, 간호사는 대상자가 가진 다양한 문제를 해결하기 위해 자신의 지식과 정보를 적극적으로 활용하여 대처할 수 있어야 한다. 따라서 예비 간호사인 대학생 시기부터 이러한 구조적인 문제해결과정을 내재화하고 실무에 적용할 수 있는 능력을 배양하는 것이 중요하다[7].

이러한 문제해결의 기초가 되는 핵심역량은 디지털 리터러시이다[8]. 디지털 리터러시는 단순히 기기 조작 능력을 넘어 디지털 환경에서 요구되는 정보를 탐색하고 평가하며 실무 상황에 적용할 수 있는 능력이다[9,10]. 특히, 다양한 보건의료 데이터를 기반으로 의사결정을 수행해야 하는 임상현장에서 디지털 리터러시는 전문직 간호사로서의 핵심역량으로 간주된다[11]. 선행연구에 따르면, 간호대학생의 디지털 리터러시는 자기주도학습[12], 문제해결능력[13], 그리고 근거기반 실무역량[6]에 영향을 미치는 요인으로 나타났다. 특히, 간호대학생을 대상으로 실시한 연구[13]에서 디지털 리터러시가 문제해결능력에 직접적인 영향을 미치는 것으로 나타났는데, 이는 디지털 리터러시가 문제해결과정에 중요한 요인임을 시사한다.

한편, 디지털 환경에서 확보한 정보를 전문적으로 통합·활용하는 능력은 간호정보활용역량으로 설명될 수 있다. 디지털 의료환경에서 의료의 질 향상과 환자 안전 개선을 위해 간호정보활용역량은 간호사의 전문적인 역할 수행을 위한 필수적인 역량으로 강조되고 있다[14]. 또한, 한국간호교육평가원이 간호학과의 주요한 학습성과 중 하나로 ‘정보통신과 최신 보건의료기술 활용’을 제시하며 간호정보역량에 대한 중요성과 체계적인 교육의 필요성을 강조하고 있다[15]. 간호정보활용역량은 간호사로서 전문직 기준과 규정을 준수하며 ICT (information and communication technology)를 활용하여 간호수행 과정에서의 정보 통합을 지원하는 능력을 의미한다[16]. 이는 현대 의료환경에서 간호사가 데이터를 체계적으로 관리하고 이를 근거로 근거기반 의사결정을 내리는 데 중요한 역할을 하고 있다[17]. 선행연구에 따르면, 간호정보활용역량은 문제해결능력뿐만 아니라 임상수행능력, 의사소통능력과도 관련이 있으며[18], 간호사의 업무수행능력[2] 및 근거기반실무역량[6] 등 다양한 간호업무성과를 높이는 핵심 요인으로 나타났다.

선행연구에서 디지털 리터러시와 간호정보활용역량이 간호대학생의 문제해결과정에 유의한 영향을 미치는 요인으로 확인되었다[13,18]. 이러한 관계를 간호정보학의 대표적인 이론적 기틀인 자료-정보-지식-지혜의 연속체(Data-Information-Knowledge-Wisdom continuum, DIKW)에 적용해 볼 수 있다. DIKW에 따르면[19,20], 개인은 단편적인 자료를 수집·조직하여 정보로 전환하고, 이를 전문적 지식체계와 결합함으로써 지식으로 발전시키며 최종적으로 이러한 지식을 실제 상황에 통합·적용하는 지혜의 단계에 이르게 된다. 이러한 관점에서 볼 때, 디지털 리터러시는 디지털 환경에서 자료와 정보를 탐색·평가하는 기초 역량으로 이해될 수 있으며, 간호정보활용역량은 확보된 정보를 간호전문직의 기준과 임상적 맥락에 맞게 통합하여 의미있는 지식으로 전환하는 전문적 역량으로 볼 수 있다. 문제해결과정은 이러한 통합된 지식을 실제 간호상황에 적용하여 최적의 의사결정에 이르는 단계로 해석할 수 있다. 즉, 디지털 리터러시는 간호정보활용역량의 기반이 되며, 간호정보활용역량은 확보된 정보가 실질적인 문제해결로 확장되는 과정을 설명하는 개념으로 이해할 수 있다. 이러한 관점은 디지털 리터러시가 문제해결과정에 영향을 미치는 과정에서 간호정보활용역량이 매개적 역할을 수행할 것으로 예측할 수 있다. 그러나 선행연구[13,18]의 대부분은 각 변수가 문제해결과정에 미치는 직접적인 영향만을 확인하는 데 그치고 있다. 즉, 간호대학생의 디지털 리터러시가 어떠한 과정을 통해 문제해결과정으로 확장되는지에 대한 연구는 부족한 실정이다.

이에 본 연구는 간호대학생의 디지털 리터러시가 문제해결과정에 미치는 영향을 확인하고, 이 관계에서 간호정보활용역량의 매개효과를 검증하고자 한다. 이를 통해 변화하는 보건의료환경에 부합하는 간호 교육과정의 방향성을 제시하고, 문제해결과정을 향상시키기 위한 기초자료를 제시하고자 한다.

### 2. 연구의 목적

본 연구는 간호대학생의 디지털 리터러시가 문제해결과정에 미치는 영향을 확인하고, 이 관계에서 간호정보활용역량의 매개효과를 검증하고자 한다. 이를 위한 구체적인 연구 목적은 다음과 같다.

첫째, 연구대상자의 디지털 리터러시, 문제해결과정, 간호정보활용역량의 수준을 파악한다.

둘째, 연구대상자의 일반적 특성에 따른 문제해결과정의 차이를 분석한다.

셋째, 디지털 리터러시, 문제해결과정, 간호정보활용역량 간의 상관관계를 분석한다.

넷째, 디지털 리터러시가 문제해결과정에 미치는 영향에서 간호정보활용역량의 매개효과를 검증한다.

## 연구 방법

### 1. 연구 설계

본 연구는 간호대학생의 디지털 리터러시가 문제해결과정에 미치는 영향을 확인하고, 이 관계에서 간호정보활용역량의 매개효과를 검증하기 위한 횡단적 서술적 조사연구이다. 본 연구는 횡단적 관찰연구의 보고지침인 Strengthening the Reporting of Observational Studies in Epidemiology (STROBE) 지침에 따라 보고하였다.

### 2. 연구 대상

본 연구의 대상자는 제주시 제주한라대학교 간호학과에 재학 중인 1–4학년 대학생으로, 편의표집을 통해 모집하였다. 연구자는 간호학과 교수로 연구대상 학년 중 일부 교과목을 담당하고 있어 연구자와 대상자 간 위계적 관계가 존재할 수 있음을 고려하여, 연구자가 개별 학생에게 직접 참여를 요청하거나 설문지를 배부·회수하지 않는 방식으로 자발성과 익명성을 보장하였다. 연구의 목적을 이해하고 자발적으로 참여 의사를 밝힌 학생을 대상으로 하였다. 적정 표본 수는 G*Power version 3.1.9.4 프로그램을 이용하여 산출하였다. 매개효과 검증을 위한 다중회귀분석 기준으로 유의수준 .05, 중간 효과크기 .15, 검정력 .80으로 설정하였다. 연령은 선행연구에서 문제해결과정과의 관련성이 일관되게 보고되지 않아 예측변수에서 제외하였으며, 회귀모형에 투입된 예측변수 7개(디지털 리터러시, 간호정보활용역량, 성별, 학년, 전공만족도, 디지털 교과목 이수 여부, 컴퓨터 활용능력)를 기준으로 산출한 결과, 필요한 최소 표본 수는 103명이었고 탈락률 20%를 고려하여 124명을 대상으로 하였다.

### 3. 연구 도구

연구에 활용된 각 도구는 개발자 및 번안자의 동의를 받은 후 연구에 적용하였다. 연구에 사용된 설문 도구는 일반적 특성 6문항, 문제해결과정 30문항, 간호정보활용역량 25문항, 디지털 리터러시 23문항으로 구성되었다.

#### 1) 일반적 특성

일반적 특성은 성별, 연령, 학년, 전공만족도, 디지털 교과목 이수 여부, 컴퓨터 활용능력 등 총 6문항으로 구성하였다. 이 중 디지털 교과목 이수 여부는 간호정보학, 인공지능개론 등 디지털 기술 및 정보활용과 관련된 교과목의 이수 경험을 의미한다. 일반적 특성 중 연령은 대상자의 특성을 기술하기 위한 목적으로 조사하였으며, 본 연구의 매개모형 및 집단 간 차이 분석에는 포함하지 않았다.

#### 2) 문제해결과정

문제해결과정은 Lee 등[5]이 개발한 문제해결과정 측정 도구를 사용하였다. 해당 척도는 5개 영역, 30문항으로 구성되어 있으며, 문제의 명료화, 해결방안 모색, 의사결정, 해결책 수행, 평가 및 반영의 하위영역으로 이루어져 있다. 각 문항은 5점 Likert 척도로 점수가 높을수록 문제해결과정 수준이 높음을 의미한다. 도구의 신뢰도 Cronbach’s α 계수를 통해 산출하였으며, Lee 등[5]의 연구에서 .93이었으며 본 연구에서는 .91이었다.

#### 3) 간호정보활용역량

간호정보활용역량은 Jo와 Ha [21]가 개발하고 Jo와 Gu [22]가 수정·보완한 도구를 활용하여 측정하였다. 본 도구는 총 25문항, 7개 하위 구성요소로 이루어져 있다. 각 문항은 5점 Likert 척도로 점수가 높을수록 간호정보활용역량이 높은 것으로 해석한다. 도구의 신뢰도 Cronbach’s α 계수를 통해 산출하였으며, Jo와 Gu [22]의 연구에서 .93이었으며, 본 연구에서는 .92로 확인되었다.

#### 4) 디지털 리터러시

본 연구에서는 Kim 등[23]의 문항을 기반으로 Wang [8]이 수정한 디지털 리터러시 측정도구를 사용하였다. 이 도구는 총 23문항으로 이루어져 있으며, 각 문항은 5점 Likert 척도로 평가하며 점수가 높을수록 디지털 리터러시 수준이 높음을 의미한다. 도구의 신뢰도는 Cronbach’s α 계수를 통해 산출하였으며, Wang [8]의 연구에서 .92였으며 본 연구에서도 .92로 나타났다.

### 4. 자료 수집

자료수집은 2025년 11월 25일부터 12월 20일까지 이루어졌다. 대상자 모집은 간호학과 게시판에 연구 모집 공고문을 게시하는 방식으로 이루어졌다. 공고문에는 연구의 목적과 절차, 예상 소요시간, 익명성 보장, 연구 참여의 자발성 및 철회 가능성, 1인 1회 참여 가능에 대한 내용을 포함하였다. 연구 참여를 원하는 학생은 학생 휴게실에 비치된 연구설명문을 충분히 읽고 이해한 후 자발적으로 동의서를 작성하였으며, 동의서와 설문지는 보관함에 직접 제출하도록 하여 익명성을 보장하였다. 총 124부를 배부하여 122부가 회수(회수율 98.4%)되었다. 이 중 설문 전체 문항의 10% 이상 무응답이거나 주요 변수(디지털 리터러시, 간호정보활용역량, 문제해결과정) 문항 중 1개 이상 미응답인 자료 3부를 제외하고 최종 119부를 분석에 사용하였다.

### 5. 자료 분석

수집된 자료는 SPSS version 25.0 (IBM Corp., Armonk, NY, USA) 프로그램과 PROCESS macro version 4.2를 활용하여 분석하였다. 대상자의 일반적 특성은 평균과 표준편차로 분석하였으며, 대상자의 일반적 특성에 따른 문제해결과정의 차이는 independent t-test, one-way analysis of variance로 검정하였다. 대상자의 문제해결과정, 디지털 리터러시, 간호정보활용역량 간의 관계는 Pearson 상관계수로 확인하였다. 대상자의 디지털 리터러시, 간호정보활용역량이 문제해결과정에 미치는 영향을 분석하기 위해 다중회귀분석을 실시하였다. 간호정보활용역량의 매개효과는 PROCESS macro version 4.2의 Model 4(단순매개모형)를 이용하여 매개분석을 실시하였다. 매개효과의 통계적 유의성 검증은 5,000회 Bootstrap을 통해 산출한 95% 신뢰구간을 기준으로 판단하였다.

### 6. 윤리적 고려

본 연구는 제주한라대학교 기관생명윤리위원회 승인을 받은 후 진행되었다(IRB NO. 1044348-20250516-HR-002-01). 대상자에게 연구설명문을 통해 연구의 목적과 절차, 익명성 보장, 연구 참여의 자발성 및 중도 철회 가능성, 참여 거부 시 학업이나 성적에 불이익이 없음을 서면으로 안내하였다. 연구 참여를 희망하는 학생에 한하여 자발적으로 서면 동의서를 받은 후 자료를 수집하였다. 연구 참여에 대한 별도의 금전적 보상은 제공하지 않았으며, 모든 자료는 익명으로 처리하여 개인정보 보호를 유지하였고, 연구 목적 범위 내에서만 활용하였다.

## 연구 결과

### 1. 대상자의 일반적 특성

대상자의 73.1%가 여학생이었으며, 평균 연령은 23.4세였다. 학년은 1학년 22.7%, 2학년 26.9%, 3학년 24.3%, 4학년 26.1%였다. 전공만족도는 만족이 50.4%로 많았으며, 디지털 교과목을 이수한 경우가 73.9%로 나타났으며, 컴퓨터 사용 능력은 보통이 53.8%로 가장 많았다(Table 1).

### 2. 대상자의 디지털 리터러시, 문제해결과정, 간호정보활용역량 정도

대상자의 디지털 리터러시는 5점 만점에 평균 4.18 ± 0.47점으로 나타났으며, 문제해결과정은 5점 만점에 평균 3.93 ± 0.54점, 간호정보활용역량은 5점 만점에서 평균 3.98 ± 0.49점으로 확인되었다(Table 2).

### 3. 대상자의 일반적 특성에 따른 문제해결과정 간의 차이

대상자의 일반적 특성에 따른 문제해결과정의 차이는 다음과 같다(Table 1). 성별, 학년, 전공만족도, 디지털 교과목 이수 여부, 컴퓨터 사용 능력과 문제해결과정 간의 유의한 차이는 없었다.

### 4. 디지털 리터러시, 문제해결과정, 간호정보활용역량 간의 상관관계

디지털 리터러시, 문제해결과정 및 간호정보활용역량 간의 상관관계를 분석한 결과, 디지털 리터러시는 문제해결과정(r = .63, *p* < .001) 및 간호정보활용역량(r = .81, *p* < .001)과 각각 유의한 양의 상관관계가 나타났다. 또한, 문제해결과정은 간호정보활용역량(r = .65, *p* < .001)과 유의한 양의 상관관계가 있었다(Table 3).

### 5. 디지털 리터러시가 문제해결과정에 미치는 영향에서 간호정보활용역량의 매개효과

간호대학생의 디지털 리터러시가 문제해결과정에 미치는 영향에서 간호정보활용역량의 매개효과에 대한 결과는 다음과 같다(Table 4). 회귀분석 가정을 검토한 결과, 잔차의 정규성은 히스토그램과 정규확률도(Normal P-P plot)를 통해 확인하였으며 정규분포에 근접한 것으로 나타났다. 선형성 및 등분산성은 표준화 예측값과 표준화 잔차의 산점도를 통해 확인하였으며 특정한 패턴 없이 무작위로 분포하여 가정을 충족하였다. 다중공선성은 공차한계와 분산팽창지수를 통해 검토하였으며, 공차한계는 0.33로 0.1 이상이었고 분산팽창지수는 2.98로 10을 넘지 않아 독립변수 간 다중공선성은 없는 것으로 판단하였다. Durbin-Watson 값은 1.87로 산출되었으며, 기준값인 2에 근접하여 잔차 간 자기상관은 없는 것으로 판단되었다. 또한, 이상치 여부는 Cook’s distance와 표준화 잔차를 통해 확인하였으며, Cook’s distance는 1.0미만이었고 표준화 잔차는 ± 3 이내로 나타나 영향치는 없는 것으로 판단되었다. 결측치는 완전응답 사례분석을 적용하였다.

1단계 회귀분석에서 디지털 리터러시는 간호정보활용역량에 통계적으로 유의한 영향을 미치는 것으로 나타났으며(β = .81, *p* < .001), 2단계에서는 디지털 리터러시가 문제해결과정에 유의한 영향을 미쳤다(β = .63, *p* < .001). 3단계에서는 디지털 리터러시와 간호정보활용역량을 동시에 투입하여 문제해결과정에 대한 영향을 분석하였다. 그 결과 간호정보활용역량(β = .41, *p* = .001)과 디지털 리터러시(β = .29, *p* = .013) 모두 문제해결과정에 유의한 설명변수로 확인되었다.

**Figure 1.**
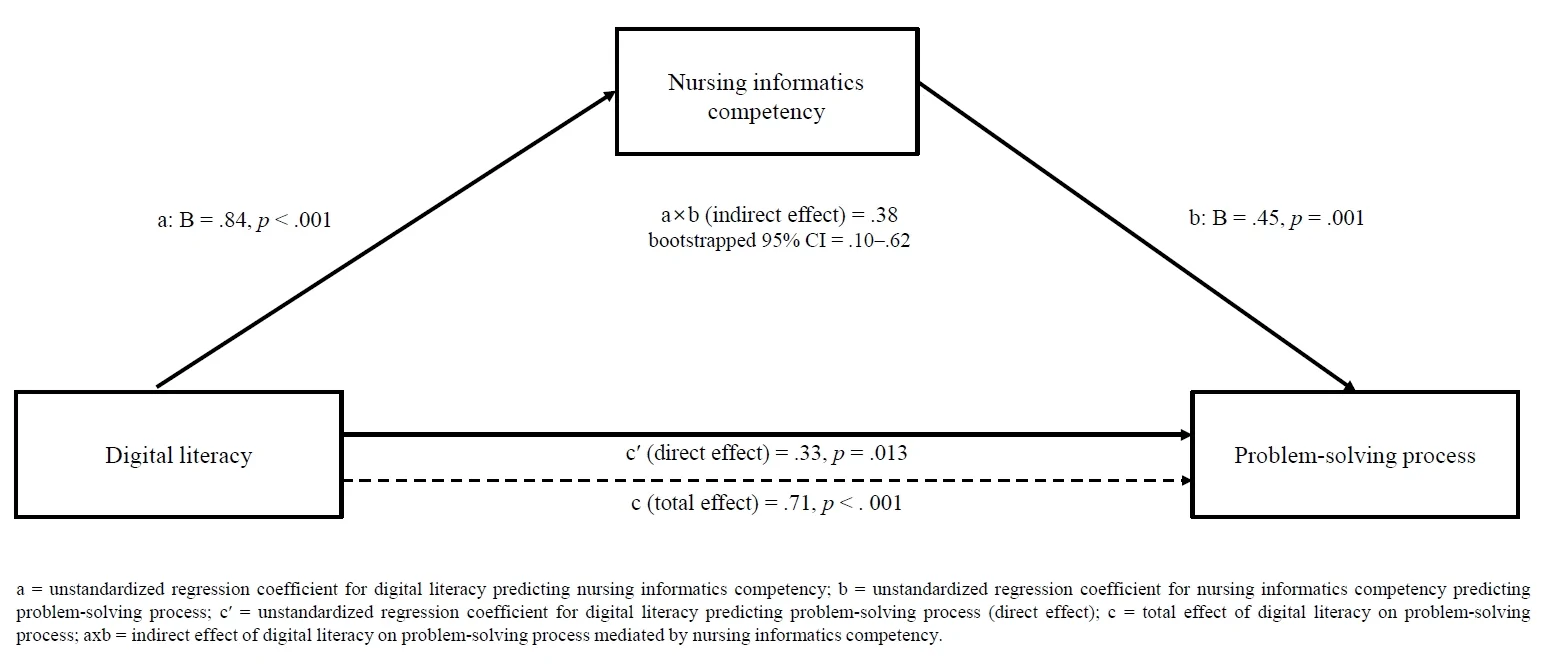
Statistical relationships in the mediation model among digital literacy, nursing informatics competency, and problem-solving process.

매개효과의 통계적 유의성을 검증하기 위해 5,000회의 bootstrap 분석을 실시한 결과(Table 4), 디지털 리터러시가 문제해결과정에 미치는 간접효과는 B = 0.38이었으며, 95% bootstrap 신뢰구간(0.10–0.62)에 0이 포함되지 않아 통계적으로 유의하였다. 또한 디지털 리터러시와 문제해결과정 간 직접효과도 통계적으로 유의하여(B = 0.33, *p* = .013), 간호정보활용역량을 통한 간접효과와 직접효과가 모두 유의한 것으로 확인되었다(Figure 1).

## 논의

본 연구는 간호대학생의 디지털 리터러시가 문제해결과정에 미치는 영향을 확인하고, 이 관계에서 간호정보활용역량의 매개효과를 검증하고자 수행되었다.

본 연구 대상자의 디지털 리터러시 수준은 5점 만점에 평균 4.18점으로 나타났다. 이는 동일 도구를 사용한 Kim 등[24]의 4.15점과 유사하나, Kim [25]의 4.05점보다 높았다. 이러한 결과는 코로나19 팬데믹 이후 대학 온라인 교육 체계의 고도화로 학생들의 디지털 활용 역량이 전반적으로 향상된 시대적 흐름과 관련이 있을 수 있다[26]. 아울러 해당 대학의 온라인 학습 시스템 및 디지털 교수법 도입 등 교육 환경적 특성이 긍정적인 영향을 미쳤을 가능성을 추정해 볼 수 있다. 다만, 본 연구에서는 교수법 노출 정도를 직접 변수로 측정하지 않았으므로 해석에 유의할 필요가 있으며, 향후 후속연구를 통한 검증이 요구된다. 한편, 보편적인 역량 향상 속에서도 학습자 간 개인차는 중요한 요소이므로, 학습자 개별 역량 차이를 반영한 맞춤형 지원전략을 병행하는 것이 필요하다[27].

대상자의 간호정보활용역량 수준은 5점 만점 중 평균 3.98점으로 확인되었으며, 동일한 도구를 활용하여 조사한 선행연구와 비교했을 때, 간호대학생을 대상으로 수행된 Koo와 Kang [3]의 3.76점, Jo 등[4]의 3.79점보다 높은 수준이었다. 이는 간호정보활용역량은 측정 시기 및 정보학 교육 여부에 따라 영향을 받는다는 선행연구[2] 결과를 고려할 때, 연구 간 자료수집 시점 및 연구가 수행된 대학의 교육환경 특성이 반영된 결과로 생각된다. Koo와 Kang [3]의 연구에서 인공지능 교육경험이 34.6%, Jo 등[4]의 연구에서 56.7%에 비해 본 연구의 대상자의 교육 경험률이 73.9%로 높게 나타났으며, 연구 간 대상자 특성의 차이도 영향을 미쳤을 것으로 생각된다. 다만, 본 연구가 일개 대학을 대상으로 수행되었으므로 향후 다양한 교육환경과 대상자 특성을 포함한 반복 연구를 통해 관련 요인을 보다 명확하게 규명할 필요가 있다.

문제해결과정은 5점 만점에 평균 3.93점으로 나타났다. 이러한 결과는 본 연구와 동일한 측정도구를 적용한 선행연구와 비교해 볼 때, 간호대학생 전 학년을 대상으로 한 Koo와 Kang [3]의 3.79점, 3학년과 4학년을 대상으로 한 Jeong [7]의 3.72점 및 Yu [28]의 3.82점보다 높게 나타났다. 이는 본 연구 대상의 대학이 문제중심학습을 교육과정에 적용하고 있을 뿐 아니라, 실제 임상 상황을 재현한 시뮬레이션실습과 AI 기반 교수법을 도입하여 학생들이 다양한 방식으로 비판적 사고와 문제해결방안을 탐색하는 경험 축적이 문제해결과정 향상에 긍정적으로 작용했을 가능성을 시사한다. 이러한 결과는 문제중심학습과 시뮬레이션 교육 적용 후 문제해결과정이 향상되었다는 선행연구[29,30]에 의해 부분적으로 지지된다. 다만, 본 연구의 설계는 특정 교수학습 전략의 효과를 검증하기 위한 목적이 아니므로 연구결과를 특정 교육방법의 영향으로 해석하기에는 제한이 있으며, 추후 관련 변수를 포함하여 다각적으로 검증할 필요가 있다.

문제해결과정은 대상자의 일반적 특성에 따라 유의한 차이를 보이지 않았다. 이는 전공만족도와 컴퓨터활용능력에 따라 문제해결과정에 유의한 차이가 있었다고 보고한 선행연구[28], 전공만족도, 컴퓨터활용능력, 인공지능 교육 경험에서 유의한 차이를 보고한 선행연구[3]와는 상이한 결과이다. Yu [28]의 연구에서는 3·4학년을 대상으로 하였고 Koo와 Kang [3]의 연구는 학년 분포에 대한 설명이 없어 직접 비교에는 제한이 있으나 연구 간 표본 구성 및 특성의 차이가 반영된 결과로 추측된다. 반면, 본 연구는 1학년부터 4학년까지 전 학년을 포함하여 대상자를 선정함에 따라 학년별 다양성이 결과에 영향을 미쳤을 것으로 생각된다. 또한 본 연구 대상자의 전공만족도와 컴퓨터활용능력이 전반적으로 높게 상향 평준화되어 집단 간 변별력이 낮아진 결과로 생각되어 향후 반복연구를 통해 이를 재확인해 볼 필요가 있다.

본 연구에서 간호대학생의 디지털 리터러시와 문제해결과정, 간호정보활용역량 간의 상관관계 분석에서 디지털 리터러시와 간호정보활용역량은 모두 문제해결과정과 유의한 양의 상관관계를 나타냈다. 이는 디지털 리터러시가 높을수록 문제해결과정이 향상된다는 선행연구[7]와 간호정보활용역량이 문제해결과정에 긍정적으로 작용한다는 선행연구[3,4]와 일치하는 결과이다.

디지털 리터러시는 단순한 디지털 기기 조작 능력을 넘어, 정보를 탐색·평가·적용하는 인지적 과정의 필수적 토대로 작용하며[9,10], 디지털 리터러시 수준이 높은 간호대학생은 방대한 보건의료 정보 속에서 필요한 정보를 신속하게 검색하고, 분석하여 임상 문제 해결에 활용할 수 있다[13]. 또한, 간호정보활용역량은 수집된 정보를 바탕으로 체계적인 대안을 탐색하고 합리적 의사결정을 내리는 과정을 통해 문제해결과정과 연결된다[28]. 이는 현대 간호실무에서 정보기술을 다루는 기초 능력과 이를 전문지식과 통합하는 능력이 문제해결과정의 중요한 요인임을 시사한다.

본 연구결과에서 간호대학생의 디지털 리터러시가 문제해결과정에 미치는 영향에서 간호정보활용역량의 매개효과를 검증한 결과, 디지털 리터러시와 문제해결과정 간 직접효과가 유의한 상태에서 간호정보활용역량을 통한 간접효과도 유의한 것으로 나타났다. 부트스트랩 분석 결과 간접효과는 .38로 나타났으며, 이는 총 효과의 약 53%를 설명하는 것으로 확인되었다. 즉, 간호대학생의 디지털 리터러시가 높을수록 문제해결과정이 향상될 뿐 아니라, 디지털 리터러시는 간호정보활용역량을 통해 간접적으로 문제해결과정에 영향을 미치는 것으로 해석된다.

한편, 본 연구에서 디지털 리터러시와 간호정보활용역량 간의 상관관계는 r = .81로 높게 나타났다. 이는 두 변인이 디지털 환경에서의 정보탐색 및 활용이라는 공통적 요소를 포함하고 있기 때문으로 볼 수 있다. 그러나 디지털 리터러시는 정보에 접근하고 평가하는 기초 역량인 반면, 간호정보활용역량은 임상현장에서 필요한 정보를 체계적으로 탐색·평가·관리하고 이를 간호문제에 적용하는 전문적 역량[3,28]이라는 점에서 구별된다. 디지털 리터러시를 통해 확보된 정보접근성이[13] 간호정보활용역량과 결합될 때, 단편적 정보습득을 넘어 비판적 사고에 기반한 문제해결과정 향상으로 이어질 수 있다[3,4,18]. 그럼에도 불구하고 본 연구에서 두 변인 간 상관계수가 높게 나타난 점은 측정 도구 간 문항 내용의 중복 가능성을 배제하기 어렵다는 점을 시사한다. 특히 두 변수를 동일 시점에서 자기보고식 설문으로 측정하였다는 점에서 공통방법편향(common method bias)의 가능성이 존재한다. 따라서 향후 연구에서는 확인적 요인분석을 통해 구성개념 간 변별타당도(discriminant validity)를 검증하고, 다양한 자료원 활용 또는 종단적 연구 설계를 통해 변수 간 관계를 보다 엄밀히 확인할 필요가 있다.

따라서 디지털 전환이 가속화되는 보건의료환경에서 간호대학생의 문제해결과정을 향상시키기 위해서는 디지털 리터러시 함양을 기반으로, 간호 관련 정보를 탐색·분석하고 이를 실제 간호상황에 적용할 수 있는 간호정보활용역량을 통합적으로 강화하는 교육적 접근이 필요하다. 선행연구[31]에서도 교육을 통해 간호정보활용역량이 향상될 수 있음이 보고된 바 있으므로 임상 실무와 연계한 실습 중심의 프로그램이나 인공지능(AI) 기반 디지털 환경에 적합한 교육과정의 설계 방안이 필요하다. 또한, 대학 차원의 보편적 인프라 구축과 더불어 학습 속도가 느린 학습자나 성인 학습자의 특성을 고려한 개별화된 교육과정 운영[27]을 병행한다면 디지털 리터러시를 보다 효과적으로 향상시킬 수 있는 방안이 될 것으로 생각된다.

본 연구 결과, 디지털 리터러시는 문제해결과정에 직접적인 영향을 미칠 뿐 아니라, 간호정보활용역량을 통한 간접적인 영향도 유의한 것으로 확인되었다. 이는 기초간호학 교육 측면에서 단순 지식 전달을 넘어, 학생들이 기초간호학 분야의 복잡한 정보와 최신 근거를 디지털 환경에서 탐색·평가하고 이를 간호문제 해결에 적용하는 교육전략이 필요함을 시사한다. 특히, 기초간호학 교과목 수업 설계 시 디지털 자료 탐색과 근거 확인 과정을 포함하는 교수학습 설계가 요구되며, 토론이나 온라인 자료 활용 과제를 통해 학생들에게 디지털 리터러시와 간호정보활용역량을 적극적으로 발휘하여 기초간호 지식을 효과적으로 수집·분석하고 이를 보건의료 문제해결과정으로 확장하는 경험을 제공할 필요가 있다. 따라서 기초간호학 교과목에서 디지털 리터러시 및 간호정보활용역량과 문제해결중심 학습을 통합적으로 운영하는 교육과정 개발이 필요할 것으로 생각된다.

본 연구의 제한점은 다음과 같다. 첫째, 일개 지역 내 간호학과 재학생을 대상으로 수행된 서술적 조사연구로, 결과를 일반화하는 데에는 제한점이 있다. 둘째, 디지털 리터러시와 간호정보활용역량 간 상관계수가 .81로 높게 나타나 두 변인 간 문항 내용의 중복 가능성과 공통방법편향의 가능성을 배제하기 어려우므로 매개효과 분석 결과의 해석에 신중을 기할 필요가 있다. 셋째, 본 연구는 대상자의 연령, 교육과정 이수 내용, 임상실습 경험, 학업성취도 등 문제해결과정에 영향을 미칠 수 있는 관련변인을 통제하지 않았다. 이는 앞서 논의한 바와 같이 대상 대학의 디지털 전환 교육환경이나 문제중심학습, 시뮬레이션 교육 경험이 연구결과에 미쳤을 가능성과도 연결되는 제한점으로, 후속연구를 통해 관련요인을 직접 측정하거나 통제한 다각적 검증이 필요하다.

## 결론

본 연구는 간호대학생을 대상으로 디지털 리터러시가 문제해결과정에 미치는 영향에서 간호정보활용역량의 매개효과를 검증하고자 수행되었다. 연구 결과, 디지털 리터러시는 문제해결과정에 직접적으로 유의한 상태에서, 간호정보활용역량을 통한 간접효과 또한 유의한 것으로 확인되었다.

따라서 간호대학생의 문제해결과정을 향상시키기 위해서는 디지털 리터러시와 간호정보활용역량을 통합적으로 강화할 수 있는 교육적 접근이 필요하다. 추후 연구에서는 문제해결과정 향상을 위한 프로그램을 개발하고 적용하는 연구를 제언한다. 또한, 다양한 학습자 특성 변인과 교육환경 요인을 포함한 확장 모형을 통해 구조적 관계를 분석하는 후속연구가 필요하다.

## Figures and Tables

**Table 1.** General Characteristics of Participants and Differences in Problem-solving Process According to General Characteristics (N = 119)

Variable	Categories	n (%) or M ± SD	Problem-solving process		
			M ± SD	t/F	*p*
Sex	Men	32 (26.9)	3.83 ± 0.56	−1.24	.216
	Women	87 (73.1)	3.97 ± 0.53		
Age (years)		23.4 ± 4.09			
Grade	1st	27 (22.7)	3.79 ± 0.46	2.48	.065
	2nd	32 (26.9)	3.83 ± 0.55		
	3rd	29 (24.3)	3.93 ± 0.61		
	4th	31 (26.1)	4.13 ± 0.46		
Satisfaction with major	Satisfied	60 (50.4)	3.98 ± 0.46	1.67	.192
	Neutral	47 (39.5)	3.91 ± 0.62		
	Dissatisfied	12 (10.1)	3.67 ± 0.54		
Completion of digital course	Yes	88 (73.9)	3.93 ± 0.51	0.29	.771
	No	31 (26.1)	3.90 ± 0.61		
Computer proficiency	High	42 (35.3)	3.95 ± 0.50	1.26	.286
	Moderate	64 (53.8)	3.95 ± 0.54		
	Low	13 (10.9)	3.70 ± 0.61		

M = Mean; SD = Standard deviation.

**Table 2.** Mean Scores for Digital Literacy, the Problem-solving Process, and Nursing Informatics Competency (N = 119)

Variables	Categories	M ± SD	Possible range
Digital literacy	Total	4.18 ± 0.47	1–5
Problem-solving process	Total	3.93 ± 0.54	1–5
Nursing informatics competency	Total	3.98 ± 0.49	1–5

M = Mean; SD = Standard deviation.

**Table 3.** Correlations among Digital Literacy, the Problem-solving Process, and Nursing Informatics Competency (N = 119)

Variables	Digital literacy	Problem-solving process	Nursing informatics competency
	r (*p*)		
Digital literacy	-	.63(< .001)	.81(< .001)
Problem-solving process	-	-	.65(< .001)

**Table 4.** Mediating effects of Nursing Informatics Competency in the Relationship between Digital Literacy and the Problem-solving Process (N = 119)

Step	Path	B	SE	β	t	*p*	Adj. R²	F	*p*	Bootstrapped 95% CI				PM
										B (a×b)	Boot SE	LLCI	ULCI	
Step 1	DL→NIC (a)	0.84	0.05	.81	15.24	< .001	.66	232.51	< .001					
Step 2	DL→PSP (c)	0.71	0.08	.63	8.92	< .001	.40	79.56	< .001					
Step 3	NIC→PSP (b)	0.45	0.13	.41	3.51	.001	.45	49.84	< .001					
	DL→PSP (c’)	0.33	0.13	.29	2.53	.013								
Indirect Effect	DL→NIC→PSP (ab)									0.38	0.13	0.10	0.62	.53

No covariates were entered in this mediation analysis. DL = Digital literacy; NIC = Nursing informatics competency; PSP = Problem-solving process; SE = Standard error of unstandardized coefficient; Boot SE = bootstrap standard error; Adj = Adjusted; CI = Confidence interval; LLCI = Lower limit of the confidence interval; ULCI = Upper limit of the confidence interval; PM = Proportion mediated.
